# Structure-based development of three- and four-antibody cocktails against SARS-CoV-2 via multiple mechanisms

**DOI:** 10.1038/s41422-021-00497-7

**Published:** 2021-03-29

**Authors:** Yao Sun, Lei Wang, Rui Feng, Nan Wang, Yuxi Wang, Dandan Zhu, Xiaorui Xing, Peng Yang, Yanjun Zhang, Weimin Li, Xiangxi Wang

**Affiliations:** 1 CAS Key Laboratory of Infection and Immunity, National Laboratory of Macromolecules, Institute of Biophysics, Chinese Academy of Sciences, Beijing, 100101 China; 2 University of Chinese Academy of Sciences, Beijing, 100049 China; 3 Department of Respiratory and Critical Care Medicine, West China Medical School/West China Hospital, Sichuan University, Chengdu, Sichuan 610041 China; 4 Department of Microbiology, Zhejiang Provincial Center for Disease Control and Prevention, Hangzhou, Zhejiang 310000 China; 5 Bioland Laboratory (Guangzhou Regenerative Medicine and Health Guangdong Laboratory), Guangzhou, Guangdong 510200 China

**Keywords:** Structural biology, Immunology

Dear Editor,

The ongoing coronavirus disease 2019 (COVID-19) pandemic caused by severe acute respiratory syndrome coronavirus-2 (SARS-CoV-2) has resulted in an unprecedented public health crisis, galvanizing a global effort for rapidly developing new therapeutic strategies effective against COVID-19. Human monoclonal antibodies (mAbs) are promising therapeutic molecules that can be used for the prevention or treatment of viral infectious diseases, including COVID-19. For instance, ZMapp^TM^, a cocktail consisting of three different mAbs targeting the Ebola glycoprotein is one of the most successful antibody-based therapeutic for treating infections caused by Ebola virus.^1^ Notably, this cocktail combines the best-performing neutralizing antibodies (NAbs) screened and developed using two separate approaches, one from humanized antibodies of origin and the other from human survivors. The success of these methods indicates critical roles played by NAb diversity in the design of antibody cocktails. Cocktail therapies are not only a source of ultra-potent neutralizing activities, but they also offer advantage in overcoming potential drug resistance issues arising out of the rapid mutation of viral pathogens, in particular when selective pressure is applied. Concerningly, the emerged and rapidly spreading SARS-CoV-2 variants have arisen in the United Kingdom (UK), South Africa (SA) and other regions, such as more recently reported B.1.1.7 (UK strain, variant of concern, VOC202012/01) and 501Y.V2 (SA strain, VOC501Y.V2). These variants contain multiple mutations in their spike proteins (S), some of which are key targets of NAbs, highlighting the tremendous potential of multiple antibody-based cocktails in treating SARS-CoV-2 infection.

Numerous human NAbs against SARS-CoV-2 target the receptor-binding domain (RBD) of the S, primarily blocking the interactions between the S and its receptor ACE2.^2–6^ Recent studies have also reported antibodies that potently neutralize SARS-CoV-2 by binding to the N-terminal domain (NTD) of the S,^7^ raising the possibility of formulation of an antibody cocktail capable of targeting both the RBD and NTD.^8,9^ Here we describe parallel high-throughput efforts undertaken for the generation of a large collection of highly potent humanized/human NAbs capable of binding to both the RBD and NTD using mouse and human survivors.^2–4,8,9^ Amongst the antibodies identified during the screening, two humanized NAbs, H014 and HB27, and one fully human NAb, P17, target the RBD and confer effective protection against SARS-CoV-2 in animal models. It is worth noting that H014 exhibited cross-neutralization activity against SARS-CoV and SARS-CoV-2, while others were SARS-CoV-2 specific.^2–4^ Additionally, both HB27 and P17 exert the double-lock type of mechanism of neutralization by which they block receptor attachment and interfere with viral membrane fusion.^3,4^ FC05, an NTD-directing NAb, was demonstrated to magnify the neutralizing potency by ~500-fold with an IC_50_ value up to pM level when used in combination with individual RBD-targeting NAbs.^9^ Correlated with this, the NTD of SARS-CoV-2 has also been demonstrated to be involved in entry into host cells by targeting the high-density lipoprotein (HDL) scavenger receptor B type 1 (SR-B1) and tyrosine-protein kinase receptor UFO (AXL).^10,11^ The significantly enhanced neutralizing potency may result from the full occupancies of RBD and NTD by RBD-directing NAbs and FC05, respectively, which almost abolishes viral attachments mediated by ACE2 or AXL or SR-B1. Although several two-antibody cocktails have been reported,^6,8,12^ three- or four-antibody cocktails have not been thoroughly characterized as yet.

To explore the possibility of the formulation of a cocktail containing three or four NAbs and test its effectiveness against SARS-CoV-2, we firstly examined the simultaneous binding of the three RBD-targeting NAbs to S trimer by competitive surface plasmon resonance (SPR). The CM5 sensor labeled with SARS-CoV-2 S trimer was fully saturated with one antibody and flooded with the other two antibodies in the flow through. Full saturation of HB27 was revealed to occlude the attachment of P17 to the SARS-CoV-2 S trimer and vice versa (Supplementary information, Fig. S1). Interestingly, full occupancy of HB27 blocked the binding of H014 to the SARS-CoV-2 S trimer, whereas HB27 could still attach to the S trimer in the presence of excessive H014 (Supplementary information, Fig. S1), which is line with structural observations of 0–3 bindings of H014 to the S trimer.^2^ By contrast, H014 and P17 were demonstrated to simultaneously bind to the SARS-CoV-2 S trimer (Supplementary information, Fig. S2). As expected, the binding of the NTD-targeting FC05 does not affect the interactions between any of the three RBD-specific NAbs and the SARS-CoV-2 S trimer (Supplementary information, Fig. S3). Not surprisingly, competitive binding assays verified a rational three-antibody cocktail consisting of FC05, H014 and P17 that simultaneously target three distinct regions (Fig. 1a). In most cases, binding of one NAb causes no notable changes in the binding affinities for other NAbs (Supplementary information, Fig. S4). Interestingly, partial occupancy of the epitopes with H014 yielded ~100-fold enhanced binding affinity for HB27, indicating an allosteric mechanism by which the conformational alterations caused by H014 binding might facilitate the interaction with HB27 in a synergistic manner (Fig. 1b). Theoretically, HB27 might act as a fourth partner to constitute the multi-component cocktail under the condition of the partial saturation of the RBD with H014 or P17. These results highlight the potential of these antibodies in formulating three- and four-antibody cocktails where the antibodies work cooperatively.

**Fig. 1:**
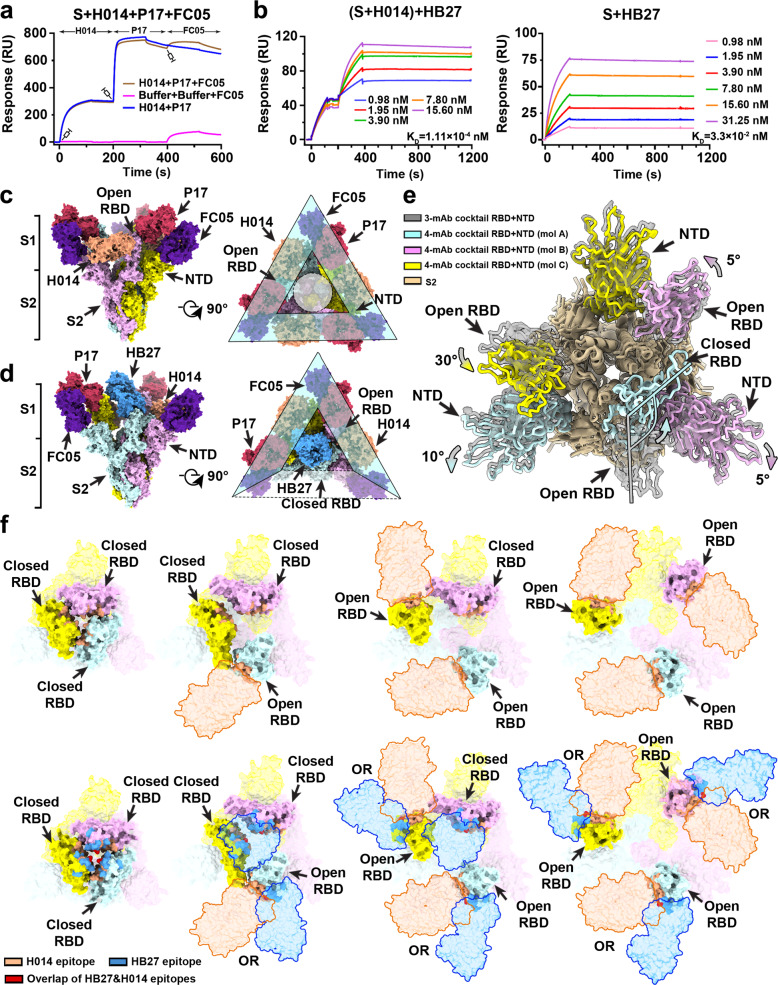
Structural basis for cooperativity observed in multiple-antibody cocktails. **a** SPR-based analysis of the competitive binding of three-antibody cocktail. **b** Binding affinities of HB27 prior to (right) or after (left) partial saturation of H014 to SARS-CoV-2 S trimer. **c**, **d** Orthogonal views of SARS-CoV-2 S trimer in complex with the 3-mAb (**c**) and 4-mAb cocktails (**d**). The S1 and S2 regions are labeled. The triangular exterior of the S trimer by nine copies of Fab molecules and the triangular interior on the top of S2 are marked by triangle and circle. **e** The conformational alterations of RBD and NTD driven by different binding patterns. **f** Top views of four possible conformations of SARS-CoV-2 S in complex with H014 (upper panel) or the mixture of H014 and HB27 (lower panel). Left to right, S trimers with 0–3 open RBDs. H014 and HB27 Fab molecules are outlined by bright colored lines. Two different binding patterns of either H014 or HB27 to the open RBDs are analyzed and marked by “OR”.

We previously reported atomic structures of the SARS-CoV-2 S trimer in complex with individual Fab fragment (FC05, H014, HB27, P17) and characterized their epitopes.^2–4,9^ The seemingly stochastic movements of the RBD give rise to two distinct conformational states referred to as the “closed” and “open” states. The corresponding epitopes in both open and closed RBDs are conditionally accessible to HB27 and P17, but accessible to H014 only in open RBDs. Interestingly, binding of H014, but not P17 or HB27, prevents closure of the RBD, leading to the significantly increased transition of the RBD from the “closed” to “open”. To precisely visualize the cooperativity of the potential three- and four-antibody cocktails, we performed cryo-EM analysis of the SARS-CoV-2 S trimer in complex with two sets of multiple-antibody cocktails (FC05–H014–P17; FC05–H014–P17–HB27) with overall resolution of 3.6 Å (Fig. 1c, d; Supplementary information, Figs. S5–7 and Table S1).

In the cryo-EM structure of the three-antibody cocktail, there are in total nine copies of Fabs bound to one S trimer, where three FC05 Fabs bind on the side of each NTD, while three H014 and three P17 Fabs bind at the side and top of each RBD, respectively, shielding the intact S1, thereby completely blocking any possible contacts with receptors and cell surface proteases (Fig. 1c). Interestingly, nine copies of Fab molecules are closely arrayed around the triangular exterior of the S trimer, tightly bridging all three S1 subunits and restraining their conformational transition, a prerequisite for viral membrane fusion (Fig. 1c). All three RBDs stand with an open configuration upon H014 binding, leaving room for the triangular interior on the top of S2 (Fig. 1c). In the cryo-EM structure of the four-antibody cocktail mixed with the S trimer, eight copies of Fab molecules, including three FC05, two H014, two P17 and one HB27, attach to one S trimer, fully occupying three vertexes and two sides of the “triangle” (Fig. 1d). Distinct from the three-antibody cocktail, two RBD domains with simultaneous bindings of H014 and P17 adopt open conformation, while the other one RBD (cyan, mol A) interacting with HB27 alone inserts into the groove constructed by the NTD and RBD from its neighboring S (violet, mol B), forcing the contacted RBD and NTD to rotate outside by ~5° (Fig. 1e). Accompanied with the transition of the RBD, sterically tight packing on the third side of the “triangle” is largely released, resulting in ~10° and 30° shifts of the NTD (cyan) from itself and the RBD (yellow) from the mol C, respectively (Fig. 1e). The conformational alterations driven by various binding patterns suggest dynamic, allosteric and synergistic effects on binding and neutralization. Although HB27 has potential to target both the open and closed RBDs, structural superimpositions demonstrate that full saturation of HB27 could possibly occur only when at least two RBDs of S trimer are in the open conformation due to the steric clashes raised by the other copies of itself in the presence of over two closed RBDs (Fig. 1f). The binding epitope on the closed RBD could only be accessed by HB27 when at least one RBD on S trimer is in the open conformation (Fig. 1f). Correlated with these, partial occupancy with H014 triggers the transition of the RBDs from the closed to open, mediates conformational changes of the unbound RBD and its surrounding microenvironments and further facilitates the binding of HB27 to the unoccupied RBD, which probably translates into the 100-fold increased binding affinity measured by SPR. Apart from the enhanced cooperativity in binding observed for this four-antibody cocktail, such a cocktail could also potentially further increase the diversity and complexity of antibodies essential for counteracting the eminent threat posed by the development of mutations. To explore the effect of the currently dominant variants on this multiple-antibody cocktail neutralization, S trimers from the B.1.1.7 and 501Y.V2 variants were expressed and evaluated for their binding affinities to these NAbs (Supplementary information, Figs. S8 and S9). Structural analysis had revealed that residues comprising the H014 epitope are identical among SARS-CoV-2 and the two dominant variants tested in this study (Supplementary information, Tables S2 and S3). In line with this analysis, H014 exhibited an indistinguishable binding affinity to S trimers from SARS-CoV-2, B.1.1.7 and 501Y.V2 (Supplementary information, Fig. S9). Interestingly, one point mutation has crept in the epitope of HB27 in B.1.1.7 and 501Y.V2. Despite this, the S trimers from SARS-CoV-2 and its two variants showed comparable binding abilities to HB27 (Supplementary information, Fig. S9, Tables S2 and S3). As expected, P17 bound to the variant B.1.1.7 as efficiently as SARS-CoV-2 since none of the RBD mutations in B.1.1.7 lies within the P17 epitope. However, binding of this antibody to the variant 501Y.V2 was reduced substantially because of the emergence of a point mutation, E484K, in the epitope. Similarly, deletion of residue 141 and presence of the new R246I mutation in the epitope of FC05 in B.1.1.7 and 501Y.V2, respectively, decreased the binding affinity of FC05 for both variants dramatically (Supplementary information, Fig. S9, Tables S2 and S3). These results indicate the usefulness of combining the neutralizing activities of different antibodies in a cocktail for countering the immune-escape threat posed by current variants, underlining the outstanding advantages of multiple NAb-based cocktails as therapeutics against SARS-CoV-2 infections.

In summary, this work dissects the nature of the cooperative interactions observed among antibodies exhibiting distinct specificities for three or four epitopes of the S trimer when mixed together in a cocktail and unveils the molecular basis of cooperative binding of the S trimer by the antibodies for synergistic neutralization and protection via multiple mechanisms. Principles of antibody–antigen interactions revealed here could also aid in the rational design of therapeutic cocktails against other viruses.

## Supplementary information

Supplementary Information

## Data Availability

Cryo-EM density maps of the FC05–H014–P17–S and FC05–H014–P17–HB27–S complexes have been deposited at the Electron Microscopy Data Bank with accession codes EMD-30993 and EMD-30994, and related atomic models have been deposited in the protein data bank under accession code 7E5R and 7E5S.
