# Supplementary material for: Structure-based development of three- and four-antibody cocktails against SARS-CoV-2 via multiple mechanisms
Source: Cell Res. 2021 Mar 29;31(5):597–600. doi: 10.1038/s41422-021-00497-7 (PMC8005859; doi:10.1038/s41422-021-00497-7)
Supplement: Supplementary file 1 — Supplementary Information [file 41422_2021_497_MOESM1_ESM.pdf]

Supplementary information

Methods and Materials

Cloning, expression and purification

The gene sequences of SARS-CoV-2 S (residues 1-1208, GenBank: MN908947.3) and two variants (B.1.1.7 and 501Y.V2) were constructed into the vector pCAGGS with a T4 fibritin trimerization motif, an HRV3C protease cleavage site and a Twin-Strep-tag in the C terminus and were mutated as previously reported [1]. The expression vectors were used to transiently transfect 293F cells (Thermo Fisher Scientific) by polyethylenimine. SARS-CoV-2 and two variant S trimers were purified from the filtered cell supernatants using StrepTactin resin (IBA). Then the eluted samples were concentrated and loaded onto a Superose 6 10/300 GL (GE Healthcare) for further purification in 20 mM Tris, 200 mM NaCl, pH 8.0. All the Fab fragments were generated using a Pierce™ Fab Preparation Kit (Thermo Fisher Scientific) according to the manufacturer's instructions.

Cryo-EM sample preparation, data acquisition and structure determination

The cryo-EM samples of SARS-CoV-2 S trimer in complex with three-antibody or four-antibody cocktail Fab fragments were prepared by quickly mixing purified SARS-CoV-2 S trimer and individual Fabs in order (FC05, H014 and P17 for three-antibody cocktail with a molar ratio of S trimer:FC05:H014:P17=1:6:6:6; FC05, H014, P17 and HB27 for four-antibody cocktail with a molar ratio of S trimer:FC05:H014:P17:HB27=1:6:3:3:3) on ice. Immediately after this, the mixtures were loaded onto the freshly glow-discharged grids (C-flat 1.2/1.3 Au). After 3 s blotting in 100% relative humidity, the grid was plunged into liquid ethane automatically by Vitrobot (Thermo Fisher Scientific). The Cryo-EM datasets were collected at 300 kV using Titan Krios microscope (Thermo Fisher Scientific) equipped with a K2 detector (Gatan, Pleasanton, CA). Movies were recorded at 32 frames, with total dose of 60 $e^{-}Å^{-2}$ and a 1.5-2.7 μm defocus using serialEM yielding the final pixel size of 1.04 Å.

Totally 1,262 micrographs for S trimer & three-antibody cocktail complex and 1,977 micrographs for S trimer & four-antibody cocktail complex were collected. RelionCorr [2] was used to correct beam induced motion and average frames. The GPU accelerated Gctf [3] was

used to estimate CTF parameters. The particles were picked automatically with Relion AutoPick. Totally 179,063 and 262,598 particles for S trimer & three-antibody cocktail complex and S trimer & four-antibody cocktail complex were picked out and further used for 2D and 3D classification, yielding a total of 92,127 and 133,167 particles for the final reconstruction. After the high-resolution refinement and postprocess in Relion, the final maps with the resolution of 3.6 Å for both of the two cocktail complexes were obtained. The resolutions were evaluated on the basis of the gold-standard Fourier shell correlation (threshold = 0.143) and the local resolution was evaluated by ResMap [4].

Structure of SARS-CoV-2 S in complex with FC05 or H014 or HB27 or P17 (Protein Data Bank ID:7D4G, 7CAH, 7CYP and 7CWO) was manually fitted into the refined EM maps using Chimera [5], corrected manually with COOT [6] and refined with phenix [7]. The final models were evaluated by Molprobity [8] and all the statistics of datasets and refinement are summarized in Table S1.

Surface plasmon resonance

SARS-CoV-2 S trimer was immobilized onto a CM5 sensor chip surface using the NHS/EDC method using Biacore T100 (GE Healthcare) and a PBS running buffer (with 0.05% Tween-20). H014, HB27, P17 and FC05 were purified for the competitive binding assays. The first antibody flew over the chip at a rate of 20 ul/min. After saturated with the first antibody, the other antibodies individually were injected at the same rate for another 120s. The data was analyzed using Biacore T100 Evaluation Software (GE Healthcare).

ELISA

The S trimer proteins of SARS-CoV-2 and two variants (B.1.1.7 and 501Y.V2) were purified and coated on 96-well plates over night at 4°C. 1% BSA was used for blocking at 37°C for 2 h. Serial dilutions of antibodies were then added and incubated at 37°C for 1 h. After washing away the unbound antibodies, HRP labelled secondary antibody against human IgG were added and incubated for another one hour. After washing away the excess secondary antibody, TMB solution was added and incubated for 5-15 min followed by addition of 1% H_2SO_4 to stop the reaction. Absorbance at 450 nm was monitored using a microplate reader.

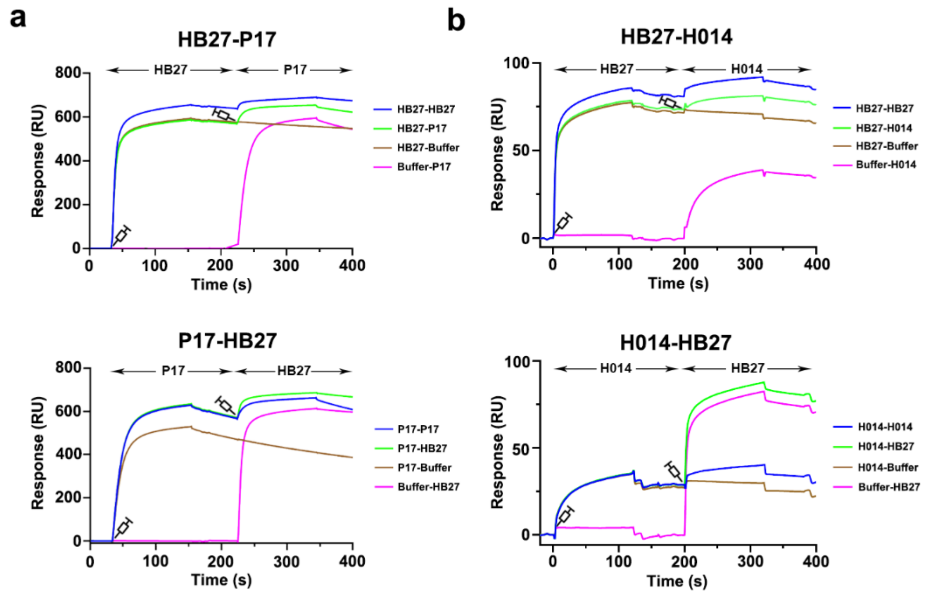

Supplementary information, Fig. S1 SPR kinetics of competitive binding of HB27 and H014 or P17. SARS-CoV-2 S was immobilized onto the CM5 sensor. HB27 was first injected, followed by P17 (a) or H014 (b); vice-versa P17 or H014 was injected first and then HB27 (lower panel).

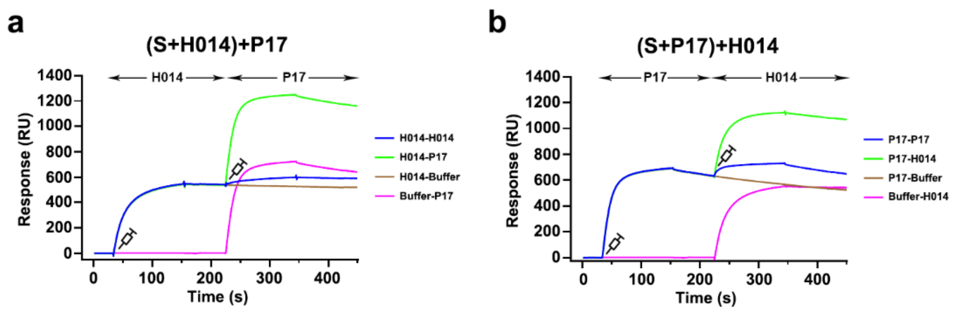

Supplementary information, Fig. S2 SPR kinetics of competitive binding of H014 and P17. SARS-CoV-2 S was immobilized onto the CM5 sensor. H014 was first injected, followed by P17 (a); vice-versa P17 was injected first and then H014 (b).

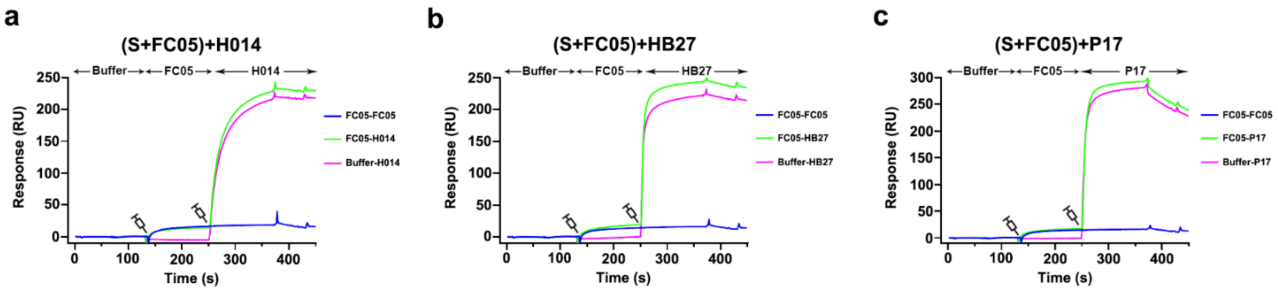

Supplementary information, Fig. S3 SPR kinetics of competitive binding of FC05 and three RBD-targeting NAbs. SARS-CoV-2 S was immobilized onto the CM5 sensor. FC05 was first injected, followed by H014 (a), HB27 (b) and P17 (c), respectively.

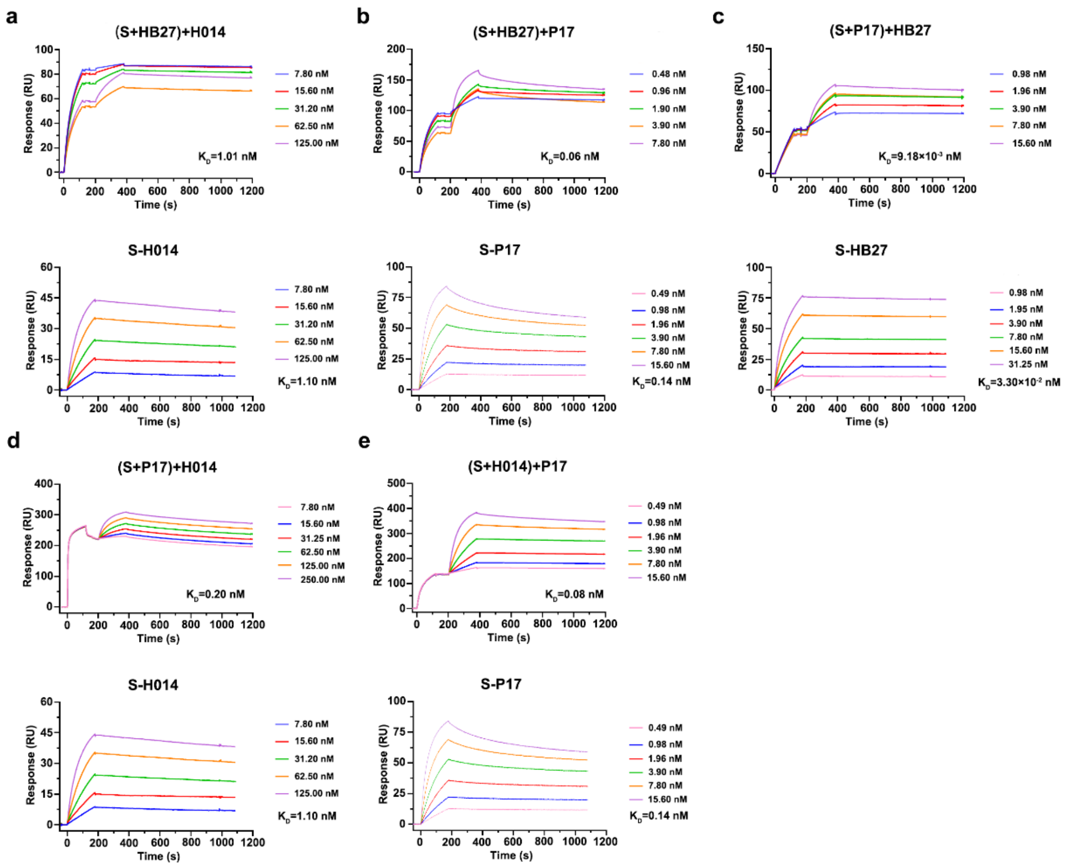

Supplementary information, Fig. S4 Binding assays for S trimer with HB27 or H014 or P17 after the first antibody was saturated by SPR. No obvious synergistic effects were observed.

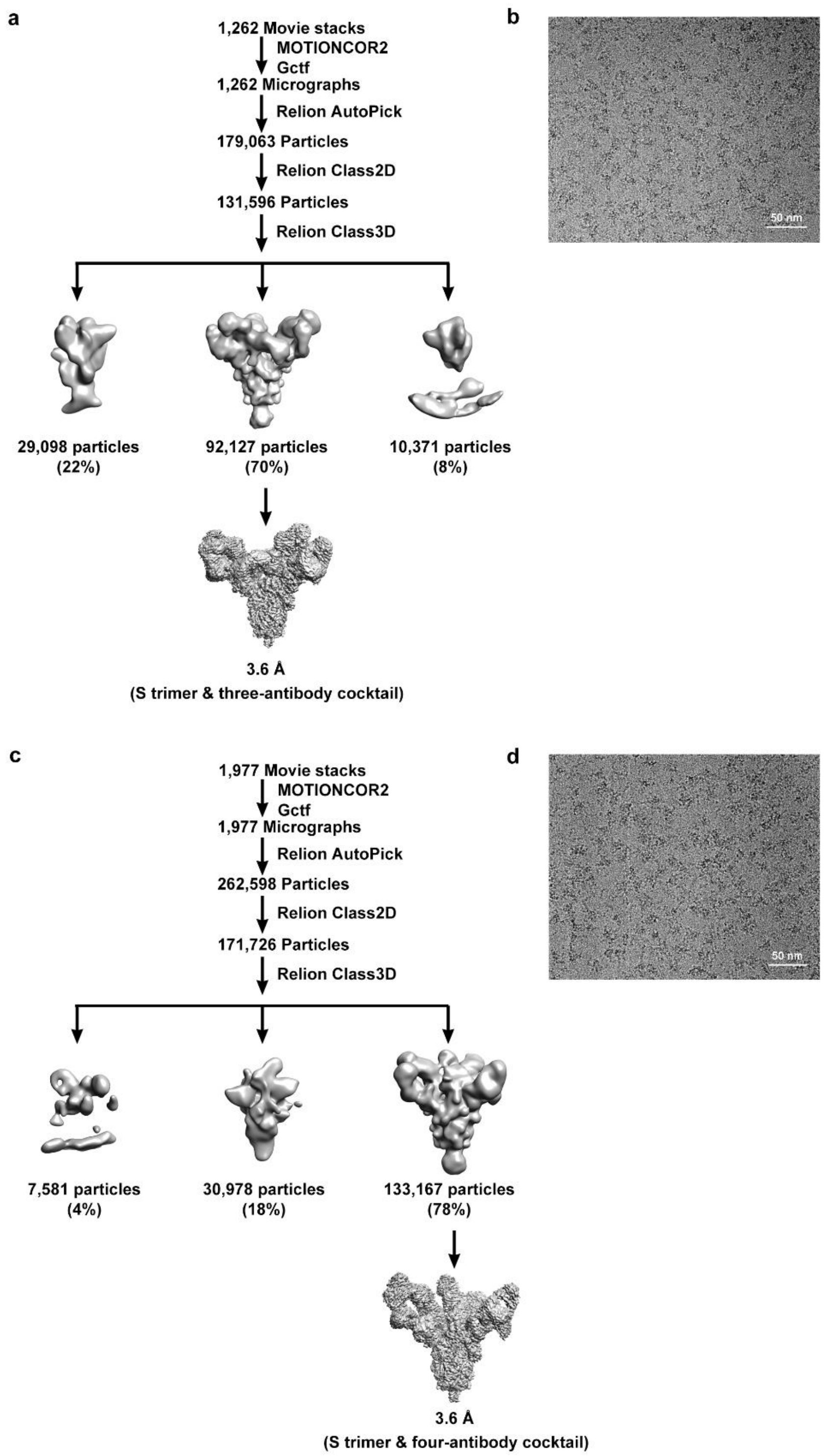

Supplementary information, Fig. S5 Flowchart for cryo-EM data processing. a Flowchart for cryo-EM data processing for three-antibody cocktail complex. **b** Cryo-EM image of three-antibody cocktail complex. **c** Flowchart for cryo-EM data processing for four-antibody cocktail complex. **d** Cryo-EM image of four-antibody cocktail complex.

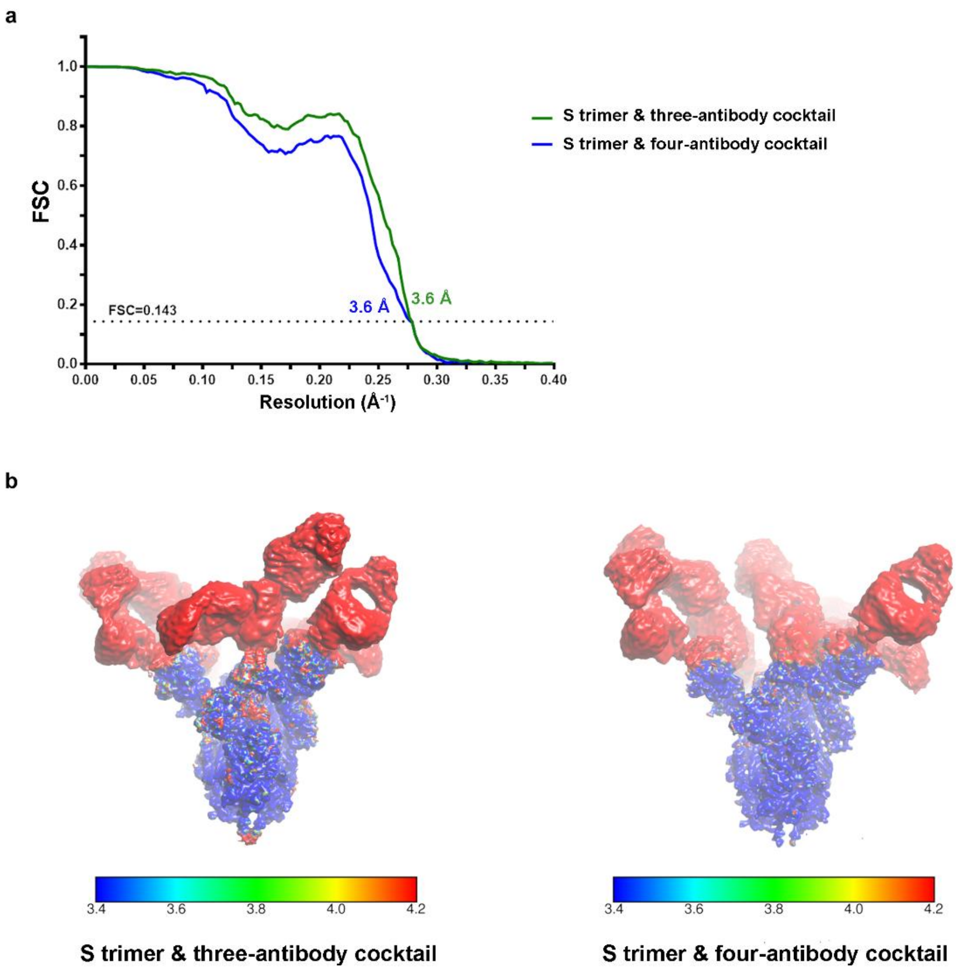

Supplementary information, Fig. S6 Validation of the cryo-EM maps. a The gold-standard FSC curves of the final maps. **b** Local resolution of the cryo-EM maps of the three- and four-antibody cocktails complexes evaluated using ResMap.

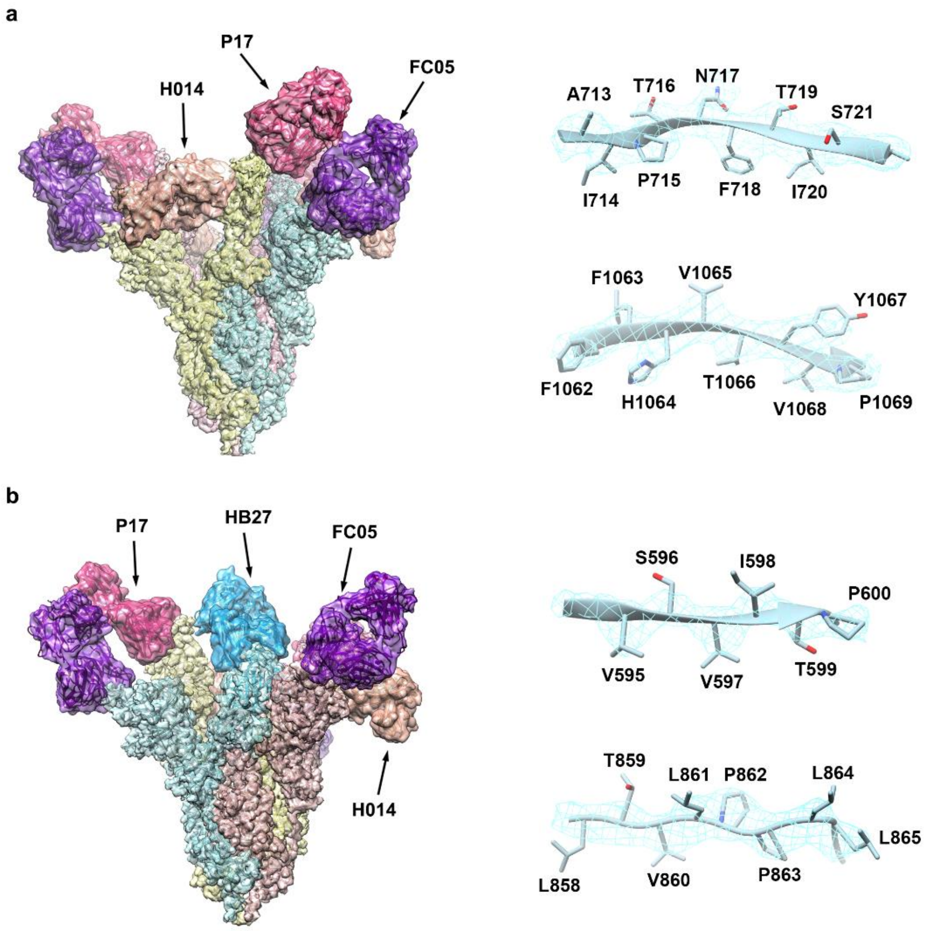

Supplementary information, Fig. S7 Density maps and atomic models. a Cryo-EM map of SARS-CoV-2 S trimer with three-antibody cocktail complex. **b** Cryo-EM map of SARS-CoV-2 S trimer with four- antibody cocktail complex. The right panels present the density maps (mesh) and related atomic models shown as sticks.

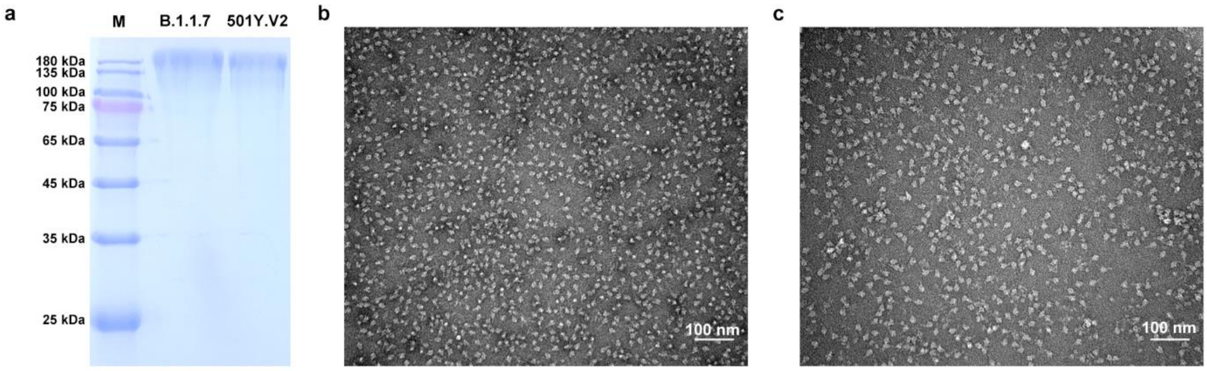

Supplementary information, Fig. S8 Characterization of SARS-CoV-2 S trimers from B.1.1.7 and 501Y.V2 variants. a SDS-PAGE analysis of the SARS-CoV-2 S trimers from B.1.1.7 and 501Y.V2. **b** Negative stain image of the SARS-CoV-2 S trimers from B.1.1.7 variant. **c** Negative stain image of the SARS-CoV-2 S trimers from 501Y.V2 variant.

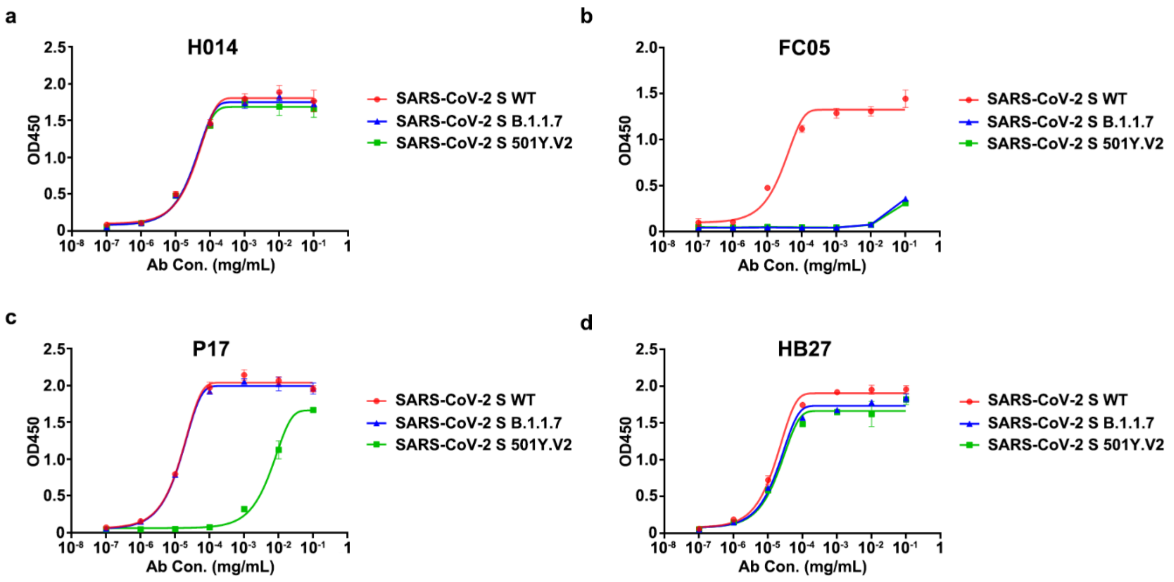

Supplementary information, Fig. S9 Binding assays of antibodies to SARS-CoV-2 S and S variants by ELISA. **a** Binding assays of H014 to SARS-CoV-2 S and S variants. **b** Binding assays of FC05 to SARS-CoV-2 S and S variants. **c** Binding assays of P17 to SARS-CoV-2 S and S variants. **d** Binding assays of HB27 to SARS-CoV-2 S and S variants.

Supplementary information, Table. S1 Cryo-EM data collection and refinement statistics

Protein	S trimer & three-antibody cocktail	S trimer & four-antibody cocktail
Voltage (kV)	300	300
Detector	K2	K2
Pixel size (Å)	1.04	1.04
Electron dose (e^{-}/Å^{2})	60	60
Defocus range (μm)	1.5-2.7	1.5-2.7
Final particles	92,127	133,167
Final resolution (Å)	3.6	3.6
**Models refinement and validation statistics**		
Ramachandran statistics		
Favored (%)	92.32	92.23
Allowed (%)	7.28	7.36
Outliers (%)	0.40	0.41
Rotamer outliers (%)	0.84	0.83
R.m.s.d		
Bond lengths (Å)	0.01	0.01
Bond angles (°)	1.27	1.27

Supplementary information, Table. S2 Effect of epitopes of NAbs in our cocktail on UK mutant strain B.1.1.7

Antibody	Epitope	del69–70	del144	N501Y	A570D	D614G	P681H	T761I	S982A	D1118H	Effect by epitope
H014	369; 372; 373; 374; 375; 376; 377; 378; 379; 380; 383; 385; 386; 405; 407; 408; 411; 412; 413; 439; 503	N	N	N	N	N	N	N	N	N	N
FC05	144; 145; 146; 147; 150; 152; 246; 247; 248; 249; 251	N	Y	N	N	N	N	N	N	N	Y
P17	455; 456; 470; 471; 481; 482; 483; 484; 485; 486; 487; 489; 490; 492	N	N	N	N	N	N	N	N	N	N
HB27	437; 440; 445; 446; 449; 499; 500; 501; 502; 503; 505; 506	N	N	Y	N	N	N	N	N	N	Y

Supplementary information, Table. S3 Effect of epitopes of NAbs in our cocktail on South Africa mutant strain 501Y.V2

Antibody	Epitope	L18F	D80A	D215G	del242-244	R246I	K417N	E484K	N501Y	D614G	A701V	Effect by epitope
H014	369; 372; 373; 374; 375; 376; 377; 378; 379; 380; 383; 385; 386; 405; 407; 408; 411; 412; 413; 439; 503	N	N	N	N	N	N	N	N	N	N	N
FC05	144; 145; 146; 147; 150; 152; 246; 247; 248; 249; 251	N	N	N	N	Y	N	N	N	N	N	Y
P17	455; 456; 470; 471; 481; 482; 483; 484; 485; 486; 487; 489; 490; 492	N	N	N	N	N	N	Y	N	N	N	Y
HB27	437; 440; 445; 446; 449; 499; 500; 501; 502; 503; 505; 506	N	N	N	N	N	N	N	Y	N	N	Y

Supplementary references

1. Wrapp, D. et al. *Science* **367**, 1260-1263 (2020).

2. Scheres, S. H. *Methods Enzymol.* **579**, 125-157 (2016).

3. Zhang, K. *J. Struct. Biol.* **193**, 1-12 (2016).

4. Kucukelbir, A., Sigworth, F. J. & Tagare, H. D. *Nat. Methods* **11**, 63-65 (2014).

5. Pettersen, E. F. et al. *J. Comput. Chem.* **25**, 1605-1612 (2004).

6. Emsley, P. & Cowtan, K. *Acta Crystallogr., Sect. D: Biol. Crystallogr.* **60**, 2126-2132 (2004).

7. Adams, P. D. et al. *Acta Crystallogr., Sect. D: Biol. Crystallogr.* **58**, 1948-1954 (2002).

8. Chen, V. B. et al. *Acta Crystallogr., Sect. D: Biol. Crystallogr.* **66**, 12-21 (2010).
